# Birdcall lures improve passerine mist-net captures at a sub-tropical African savanna

**DOI:** 10.1371/journal.pone.0199595

**Published:** 2018-06-21

**Authors:** Mduduzi Ndlovu

**Affiliations:** School of Biology and Environmental Sciences, University of Mpumalanga, Mbombela, Mpumalanga, South Africa; Princeton University, UNITED STATES

## Abstract

Field research techniques are constantly evolving to meet the needs of the scientific community. There is a growing need for field biology studies to shift towards increasing efficiency and quality of results while simultaneously decreasing cost in both the researcher’s time and resources. I tested the efficacy of using multiple recorded birdcall lures (n = 172 species) to improve mist-net captures at a subtropical African savanna setting. Capture success was compared between passive and birdcall enhanced mist-nets during winter and summer seasons. Results suggest that the use of birdcalls does significantly increase the total number of birds caught in both seasons and also increases the diversity of passerine species. Conventional passive mist-nets without an audio lure were initially productive but their capture rate subsequently decreased as sampling days progressed. Birdcall lure enhanced mist-nets had a constant capture output during the summer season. The most responsive birds to audio lures were gregarious species (e.g. *Pycnonotus barbatu*s, *Dryoscopus cubla*, *Prionops plumatus*, *Phoeniculus purpureus*, *Turdoides jardineii* and *Lamprotornis chalybaeus*) and the aggressive *Dicrurus adsimilis* and *Acridotheres tristis*. I conclude that birdcall lures can be used in summer and winter seasons to improve mist-net captures especially for studies focusing on gregarious and aggressive passerine species in a sub-tropical African savanna setting.

## Introduction

Most field biology, animal movement and wildlife health studies are dependent on direct capture and collection of samples from live free roaming animals. Recent outbreaks of emerging infectious diseases (e.g. Ebola, Avian influenza and West Nile) have also influenced the increase in surveillance studies targeting highly mobile wild animals such as bats and birds [1,2,3]. Animal capture methods have evolved from previous predominately lethal techniques to the current ethically acceptable live capture techniques. This change in wildlife research has obviously come at a cost with regards to the number of samples that can be collected from live captures. Ethics permits usually allow far more captures than collections.

Flying animals like birds and bats are generally elusive and it requires a lot of effort, time and resources for researchers to attain decent sample sizes. Use of conventional capture methods such as mist-nets can also be time consuming and little effort has been done, let alone documented, to try and improve their efficacy. Failure to adjust and improve field-sampling methods will undoubtedly impact our understanding of animal biology, ecology and emerging infectious diseases.

Studies in the northern hemisphere have employed various incentives and decoys to enhance trap productivity. Others have used recorded audio birdcalls to lure avian species into traps [4, 5, 6, 7, 8, 9]. Studies where the technique was used, it was mostly aimed at catching a single migratory bird species such as Reed Warblers *Acrocephalus scirpaceus* and Sedge Warblers *A*. *schoenobaenus* [9,10], Eurasian Skylarks *Alauda arvensis* [11]and Curlew sandpipers [12]. Most of these studies reported an increase in captures but also a significant male-bias which they attributed to the fact that males are usually more vocal than females and as such, tend to be strongly attracted by acoustic lures [9,11,13]. In other studies, e.g. in Reed warblers [9] and Wood Thrushes [14], both sexes were found to respond similarly to acoustic stimuli. To my knowledge, this method (although it may be in use) and its efficacy has never been documented for studies done in the sub-tropical African savanna and with non-migratory species.

The advancement of technology in more exciting and efficient methodologies is developing constantly, and yet science in developing countries rarely takes advantage of these improvements, let alone document its efficacy. The use of recorded birdcalls as acoustic lures for birds is simple to implement and highly effective in increasing capture numbers (and recaptures) in the northern hemisphere [4,5,6,7,8,9]. However, very little work on improving mist-net capture methodology has been established for the species diverse regions of the southern African sub-tropical savanna regions. I tested the efficacy of using recorded audio birdcalls to improve avian mist-net captures at an African subtropical savanna setting. Capture success was compared between passive and birdcall enhanced mist-nets during the winter and summer seasons to determine whether an audio lure would alter the productivity of ordinary passive mist-netting techniques. I hypothesized that the use of birdcall enhanced mist-nets would increase the numbers and diversity of birds captured.

## Materials and methods

### Ethics statement

This research was carried out in strict accordance with the recommendations and approvals given by the University of the Witwatersrand Animal Ethics Screening Committee (AESC—Clearance certificate: 2015/02/B) and South African National Parks. Sampling permits for this study were issued by the South African National Parks (the body responsible for Kruger national park) under the avian parasitology registered research project (Ref: NDLM1262). All sampling procedures and experimental manipulations were reviewed and approved as part of obtaining the field permit from Kruger national park.This research did not involve the sampling of endangered or protected species. All animals used in this research were live captures that were later released at the same site within 10 minutes from the initial capture time. All procedures and handling of birds performed during this study were in accordance with the ethical standards of the South African National Parks and the South African Ringing Scheme (SAFRING). The researcher was professionally trained and deemed competent to perform the procedures therein, in terms of Section 23(1)(c) of the Veterinary and Para-Veterinary Professions Act (19 of 1982) of South Africa and SAFRING on Animal Handling and Care. The researcher is also a registered and fully qualified ringer for all avian species in the African Continent (AFRING Ringer number:1417)".

### Study site

The study was conducted around Skukuza (24.9948° S, 31.5969° E) area inside Kruger national park (KNP). The area experiences mild (average temperature = 23°C) and dry winters whereas summers tend to be hot (average temperature = 30°C) and wet [15]. Annual rainfall ranges between 500–550 mm [15]. Mixed woodlands and pockets of grasslands dominate the area (Du Toit *et al*. 2003). The main tree species are *Acacia nigrescens*, *Acacia tortilis*, *Dichrostachys cinerea*, *Euclea divinorum*, *Spirostachys africana*, *Sclerocarya birrea* and *Ziziphus mucronata*. Approximately 200 species of passerines species occur in the southern region of the KNP around Skukuza [16].

### Fieldwork

The study was conducted during the dry winter (June) and wet summer (December) periods of 2015. Two locations with high bird activity were selected inside KNP around Skukuza area and four (3.2 m x 18 m, 4 shelves) mist-nets were used for ten-day capture trials per season. Selected trial locations were at the (1) Research Camp (24.99316° S, 31.58474° E) and (2) Skukuza Leadership Centre (24.99446° S, 31.59494° E). Two mist-nets were setup along bush trails at approximately 150 m apart at each trial location. Previous capture trials at the same locations and positions in previous years (October 2013 –April 2015) had yielded decent numbers of birds. Every day I tossed a coin to randomly assign the birdcall treatment to one of the mist-nest at each location and left the other one passive (control). The birdcall treatment was a randomly shuffled audio collection of small bushveld bird species [17] that commonly occur around the southern section of Kruger national park [16]. The playlist comprised of five *Falconidae* (Kestrels and Falcons), four near passerine (Bucerotiformes) and 162 “true” passerine (Passeriformes) species (Appendix 1). Birdcalls were played from an iPod shuffle 4^th^ generation (Apple Inc., Cupertino, California, USA) connected to a 2.5 watt power X-mini™ uno capsule speaker (Xmi Pte Ltd, Singapore). The iPod and speaker were placed underneath the mist-nest (approximately midway the length of the net), loosely covered with leaves and played at maximum volume. The same mist-net positions were maintained throughout the duration of the study. Capture trials were only done in the morning between 06h00–09h00 to coincide with the peak morning bird activity. All mist-nets were monitored during trials with the help of volunteer university students. Captured birds were identified, measured (culmen, head, tarsus, and forewing length), ringed with a uniquely numbered metal ring and immediately released.

### Data analysis

Non-parametric statistical analyses were performed since the capture data obtained was not normally distributed. A series of Wilcoxon signed-rank tests were used to compare daily passerine captures (for individual numbers and species numbers) between passive and birdcall-enhanced mist-nets for winter, summer and both seasons combined. I also used a Mann Whitney U test to determine if there was any seasonal related (dry vs wet season) differences in capture success of passive and birdcall enhanced mist-nets. To measure the rate of mist-net captures I fitted linear regression models on season captures according to treatment. All statistical analyses were carried out using the R Statistical program [18] and tested at the 5% level of significance.

## Results

**Fig 1:**
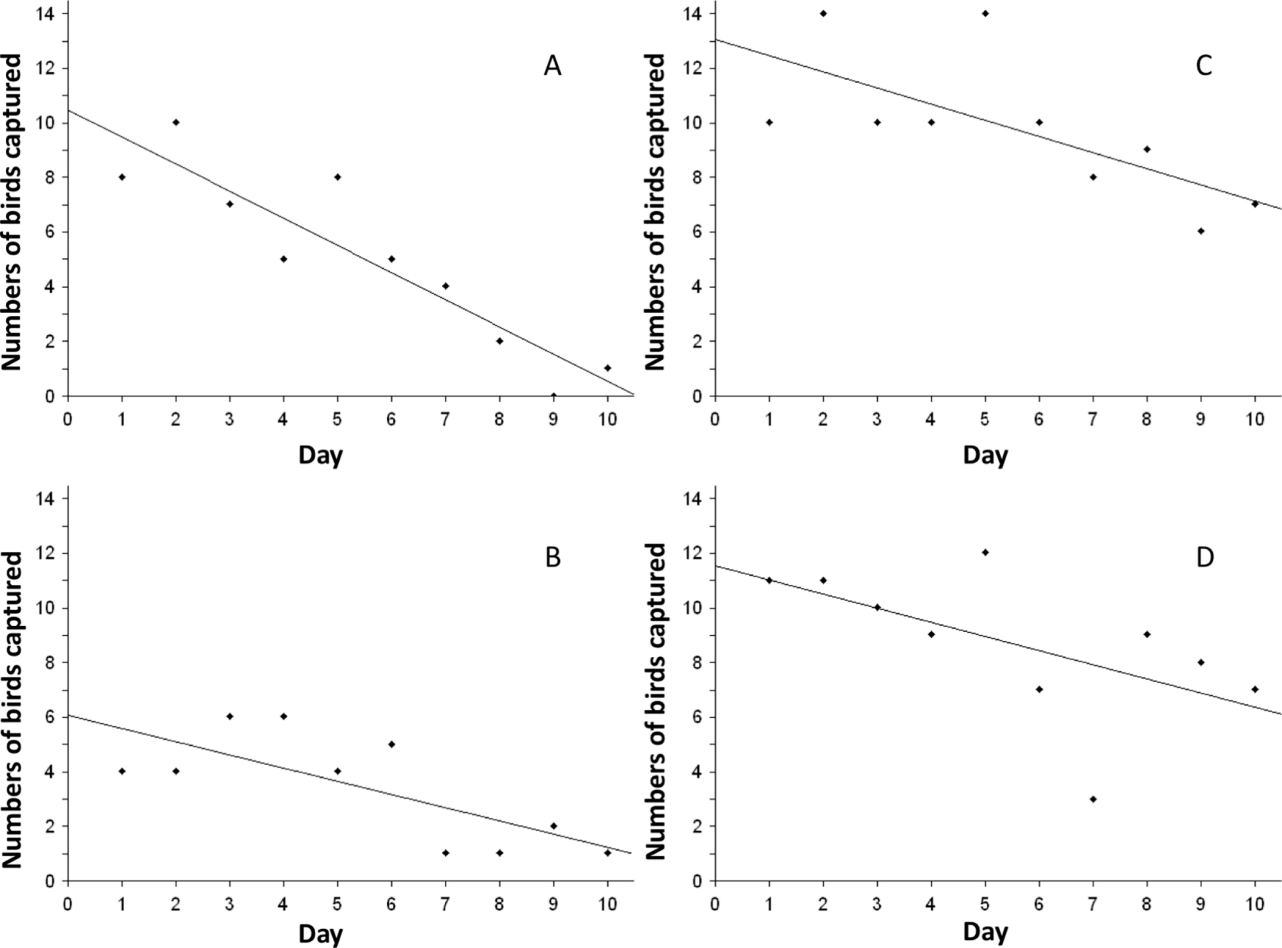
Linear regression plots of birds caught during the 10-day sampling period at Skukuza. A: Winter capture rate using passive mist-nets. B: Summer capture rate using passive mist-nets. C: Winter capture rate using birdcall enhanced mist-nets. D: Summer capture rate using birdcall enhanced mist-nets.

A total of 287 individual birds, representing 52 species were captured during the study period (S1 Appendix). All 52 passerine species (n = 203) were caught using birdcall-enhanced mist-nets and only 21 (n = 84) of those species were caught in passive mist-nets (Table 1). There were only five recaptures from four species throughout the study and recaptures were caught in birdcall enhanced mist-nets during the wet summer season (see S1 Appendix). The daily count of birds caught in passive mist-nets during the winter and summer seasons decreased (r = -0.912, p < 0.001 and r = -0.730, p = 0.017, respectively) as sampling days progressed (Fig 1). The number of birds caught in winter using birdcall-enhanced mist-nets also decreased (r = -0.687, p = 0.028) as sampling days progressed (Fig 1). However, in summer birdcall enhanced mist-nets maintained a marginally non-significant (r = -0.594, p = 0.07) capture rate throughout the sampling period (Fig 1).

**Table 1:** Numbers of birds and species count caught per day using passive and birdcall enhanced mist-nets at Skukuza during the winter and summer seasons.

Season	Day	Passive mist-net		Birdcall mist-net	
		Numbers	Species count	Numbers	Species count
Dry winter	1	8	2	10	4
	2	10	3	14	5
	3	7	4	10	4
	4	5	2	10	4
	5	8	3	14	6
	6	5	3	9	5
	7	4	2	11	6
	8	2	1	11	5
	9	0	0	13	5
	10	1	1	14	3
Wet summer	1	4	1	11	4
	2	4	1	11	6
	3	6	2	10	9
	4	6	2	9	7
	5	4	2	12	8
	6	5	3	7	4
	7	1	1	3	2
	8	1	1	9	5
	9	2	1	8	5
	10	1	1	7	2
Total	20	84		203	

Overall, a significantly higher (Wilcoxon: Z = 3.92, p > 0.001) number of birds were caught per trial using birdcall-enhanced mist-nets (Mdn = 9.5, total n = 203) as compared to passive mist nets (Mdn = 4, total n = 84). Birdcall enhanced mist-nets also yielded a higher daily diversity of bird species compared to passive mist-nets (Mdn = 5 and 2 respectively, Z = 3.82, p > 0.001). There was no seasonal difference in the numbers of birds caught using passive mist-nets (Mann Whitney U = 34.5, Z = 1.134, p = 0.257) or birds caught in birdcall-enhanced mist-nets (U = 42.5, Z = 0.529, p = 0.597). Similarly, there was also no seasonal difference in species numbers of birds caught using passive mist-nets (U = 33, Z = 1.247, p = 0.212) and also for birds caught in birdcall-enhanced mist-nets (U = 44, Z = -0.416, p = 0.678).

The most responsive birds to audio lures were gregarious species e.g. Dark-capped Bulbuls (*Pycnonotus barbatu*s), Black-backed Puffbacks (*Dryoscopus cubla*), White-crested Helmetshrikes (*Prionops plumatus*), Green wood hoopoes (*Phoeniculus purpureus*), Arrow-marked babblers (*Turdoides jardineii*) and Greater blue-eared Starlings (*Lamprotornis chalybaeus*). The aggressive Fork-tailed Drongo (*Dicrurus adsimilis*) and the Common Myna (*Acridotheres tristis*) were also attracted and mostly captured in birdcall enhanced mist-nets (see S1 Appendix). Black-crowned Tchagra (*Tchagra senegalus*), Red-capped Robin-chats (*Cossypha natalensis*), Orange-breasted Bush-shrikes (*Chlorophoneus sulfureopectus*), White-browed Robin Chat (*Cossypha heuglini*) and White-browed Scrub Robin (*Cercotrichas leucophrys*) where frequently sighted around the mist-netting area but proved elusive to catch. I only caught one of each species per season, using the birdcall enhanced mist-net.

## Discussion

My findings suggest that the use of birdcall lures does significantly increase the total number and diversity of passerine birds caught using mist-nets (Table 1). The method proved useful for recaptures even though the numbers were rather small (S1 Appendix). Most of birds that were caught in birdcall enhanced mist-nets were either gregarious (found foraging and or roasting in flocks of more that 2 individuals of the same species [19]) or territorial species. As indicated by previous studies [20,21], I also suspect that territorial birds were lured to the calls of conspecifics in an attempt to defend their territory from a perceived competitor. In contrast, more Bronze Mannikins (*Spermestes cucullata*) and Common Waxbills (*Estrilda astrild*) were caught using passive mist-nets compared to birdcall enhanced mist-nets (8 vs 4 and 7 vs 4, respectively, S1 Appendix). This may suggest that the use of birdcalls especially those of Falconidae might have deterred these smaller passerine species from the birdcall enhanced mist-nets.

Some birders who are against the use of bird calls argue that the use of birdcalls disrupts the daily activities (i.e. foraging, resting, preening etc.) of birds as they are diverted to seek or escape the perceived threat. The potentially detrimental effects of playback are subject to frequent debate in birdwatching discussion forums, where arguments against the use of recordings are frequently reported (see Sibley [22] and discussion therein). These include long lasting bird's abnormal behaviour, birds being captured by predators while attracted to the recording, or brood parasites taking advantage of the disturbance to lay their eggs in nest of distracted birds. As a result, the use of bird recordings is discouraged by several birdwatching organisations [23]. However, the scientific information on the effects of playback on wild birds is normally very scarce, and often comes as a 'by-product' of ethological studies that use playback for research purposes. Birds may show elevated levels of blood testosterone after being exposed to recordings [24], what in turn can have detrimental effects on the bird's health. Birds may also lose their social status if seen by their neighbours as 'losers' on battles against the recordings [25], and birds that become exposed by singing back to the recordings may be attacked by competitor species [26]. In some bird populations, the effects of single playback experiments carry over across several years [27]. Harris and Haskell [22] reported that the use of bird calls induced habituation to calls and changes in vocal behaviour of Plain-tailed Wrens (*Thryothorus euophrys*) and Rufous Antpittas (*Grallaria rufula*) in Ecuador. The most reported effect of the method is that it not only increases captures but causes a significant male-bias which is attributed to the fact that males tend to be more vocal than females and as such, will be strongly attracted by birdcalls [9,11,13]. There is also a possibility that the use of birdcalls may chase away other potential target species through irritation or intimidation, which would detract from sample diversity. Both aspects were not apparent in this study. I therefore recommend that future research must investigate which combinations will be most effective in both attracting an unbiased sex ratio and also not scaring away specific species.

Birdcall enhanced mist-nets also enabled the capture of normally trap elusive species (evidence from personal experience and low numbers recorded in the SAFRING database) such as the Common Myna (*Acridotheres tristis*), Long-billed Crombec (*Sylvietta rufescens*), Collared Sunbird (*Hedydipna collaris*), White-bellied Sunbird (*Cinnyris talatala*), Violet-backed Starling (*Cinnyricinclus leucogaster*), White-browed Robin-chat (*Cossypha heuglini*) and White-browed Scrub-robin (*Cercotrichas leucophrys*), hence this adjustment has the potential of increasing “physical” data on understudied species and also do so at a faster rate. For sexually dimorphic species such as the Collared Sunbird, White-bellied Sunbird and Violet-backed Starling similar numbers of both sexes were caught with no apparent male-bias.

The method is not novel (see [4,5,6,7,8,9]) but it’s use is limited especially in the African savanna settings. Perhaps the greatest challenge is that up until now researchers in the continent have not documented the advantages of using birdcall lures to enhance captures. There is also the initial extra capital required to purchase the birdcall recordings, appropriate player and portable speakers. However, that once-off investment of approximately US$100 (as of March 2017, www.amazon.com) far outweighs the benefits of improved mist-netting captures and species diversity that would have required more mist netting days and the associated fieldwork costs (i.e. time, accommodation, subsistence etc.).

Overall the findings of this study may prove particularly relevant in improving efficiency of ornithological research within the context of southern Africa savannas. This research has far-reaching potential in a variety of applications, including general collection projects, zoonotic disease monitoring, behavioural studies, and perhaps more in-depth field methodological ventures. For example, in the context of avian epidemiology surveys, we still do not fully understand how diseased birds may be influenced by playback. Further adjustments to my methodology could also significantly increase its efficacy and holds promise for prospective endeavours in the improvement of field research methodology.

## Supporting information

S1 Appendix Species list of bird-calls used in lure enhanced mist-nets. Column headings Mist-net captures (Passive / Birdcall) and Recaptured represent the numbers of birds captured per species. All five recaptures were from bird-call enhanced mist-nets.
(DOCX)
