# Supplementary material for: Birdcall lures improve passerine mist-net captures at a sub-tropical African savanna
Source: PLoS One. 2018 Jun 21;13(6):e0199595. doi: 10.1371/journal.pone.0199595 (PMC6013166; doi:10.1371/journal.pone.0199595)
Supplement: S1 Appendix — Column headings Mist-net captures (Passive / Birdcall) and Recaptured represent the numbers of birds captured per species. All five recaptures were from bird-call enhanced mist-nets. (DOCX) [file pone.0199595.s001.docx]

**S1 Appendix.**

| **Family** | **Common Name** | **Species** | **Mist-net captures** | | **Recaptured** |
| --- | --- | --- | --- | --- | --- |
|  |  |  | **Passive** | **Birdcall** |  |
| Bucerotiformes: Bucerotidae | Southern red-billed Hornbill | *Tockus rufirostris* | 2 | 3 |  |
| Bucerotiformes: Bucerotidae | Southern yellow-billed Hornbill | *Tockus leucomelas* | 3 | 4 |  |
| Bucerotiformes: Phoeniculidae | Green Wood-hoopoe | *Phoeniculus purpureus* |  | 13 | 2 |
| Bucerotiformes: Upupidae | African Hoopoe | *Upupa africana* | 2 | 1 |  |
| Falconiformes: Falconidae | Amur Falcon | *Falco amurensis* |  |  |  |
| Falconiformes: Falconidae | Common Kestrel | *Falco tinnunculus* |  |  |  |
| Falconiformes: Falconidae | Lanner Falcon | *Falco biarmicus* |  |  |  |
| Falconiformes: Falconidae | Lesser Kestrel | *Falco naumanni* |  |  |  |
| Falconiformes: Falconidae | Peregrine Falcon | *Falco peregrinus* |  |  |  |
| Passeriformes: Acrocephalidae | Common Reed Warbler | *Acrocephalus scirpaceus* |  |  |  |
| Passeriformes: Acrocephalidae | Dark-capped Yellow Warbler | *Iduna natalensis* |  |  |  |
| Passeriformes: Acrocephalidae | Icterine Warbler | *Hippolais icterina* |  |  |  |
| Passeriformes: Acrocephalidae | Marsh Warbler | *Acrocephalus palustris* | 1 | 3 |  |
| Passeriformes: Acrocephalidae | Olive-tree Warbler | *Hippolais olivetorum* |  |  |  |
| Passeriformes: Acrocephalidae | Sedge Warbler | *Acrocephalus schoenobaenus* |  |  |  |
| Passeriformes: Alaudidae | Chestnut-backed Sparrow Lark | *Eremopterix leucotis* |  |  |  |
| Passeriformes: Alaudidae | Dusky Lark | *Pinarocorys nigricans* |  |  |  |
| Passeriformes: Alaudidae | Fawn-coloured Lark | *Calendulauda africanoides* |  |  |  |
| Passeriformes: Alaudidae | Flappet Lark | *Mirafra rufocinnamomea* |  |  |  |
| Passeriformes: Alaudidae | Monotonous Lark | *Mirafra passerina* |  |  |  |
| Passeriformes: Alaudidae | Red-capped Lark | *Calandrella cinerea* |  |  |  |
| Passeriformes: Alaudidae | Rufous-naped Lark | *Mirafra africana* |  |  |  |
| Passeriformes: Alaudidae | Sabota Lark | *Calendulauda sabota* |  |  |  |
| Passeriformes: Buphagidae | Red-billed Oxpecker | *Buphagus erythrorhynchus* |  |  |  |
| Passeriformes: Campephagidae | Black Cuckooshrike | *Campephaga flava* |  |  |  |
| Passeriformes: Campephagidae | Grey Cuckooshrike | *Ceblepyris caesius* |  |  |  |
| Passeriformes: Campephagidae | White-breasted Cuckooshrike | *Ceblepyris pectoralis* |  |  |  |
| Passeriformes: Cisticolidae | Bar-throated Apalis | *Apalis thoracica* |  |  |  |
| Passeriformes: Cisticolidae | Burnt-necked Eremomela | *Eremomela usticollis* |  |  |  |
| Passeriformes: Cisticolidae | Croaking Cisticola | *Cisticola natalensis* |  |  |  |
| Passeriformes: Cisticolidae | Desert Cisticola | *Cisticola aridulus* |  |  |  |
| Passeriformes: Cisticolidae | Green-capped Eremomela | *Eremomela scotops* |  |  |  |
| Passeriformes: Cisticolidae | Grey-backed Camaroptera | *Camaroptera brachyura* |  |  |  |
| Passeriformes: Cisticolidae | Levaillant's Cisticola | *Cisticola tinniens* |  | 3 |  |
| Passeriformes: Cisticolidae | Piping Cisticola | *Cisticola fulvicapilla* |  |  |  |
| Passeriformes: Cisticolidae | Rattling Cisticola | *Cisticola chiniana* |  |  |  |
| Passeriformes: Cisticolidae | Red-faced Cisticola | *Cisticola erythrops* |  |  |  |
| Passeriformes: Cisticolidae | Rock-loving Cisticola | *Cisticola aberrans* |  |  |  |
| Passeriformes: Cisticolidae | Tawny-flanked Prinia | *Prinia subflava* |  | 4 |  |
| Passeriformes: Cisticolidae | Winding Cisticola | *Cisticola galactotes* |  |  |  |
| Passeriformes: Cisticolidae | Yellow-bellied Eremomela | *Eremomela icteropygialis* |  |  |  |
| Passeriformes: Cisticolidae | Yellow-breasted Apalis | *Apalis flavida* |  |  |  |
| Passeriformes: Cisticolidae | Zitting Cisticola | *Cisticola juncidis* |  |  |  |
| Passeriformes: Dicruridae | Fork-tailed Drongo | *Dicrurus adsimilis* |  | 11 | 1 |
| Passeriformes: Emberizidae | Cinnamon-breasted Bunting | *Fringillaria tahapisi* |  |  |  |
| Passeriformes: Emberizidae | Golden-breasted Bunting | *Fringillaria flaviventris* |  |  |  |
| Passeriformes: Estrildidae | African Firefinch | *Lagonosticta rubricata* |  |  |  |
| Passeriformes: Estrildidae | African Quailfinch | *Ortygospiza fuscocrissa* |  |  |  |
| Passeriformes: Estrildidae | Black-and-white Mannikin | *Spermestes bicolor* |  |  |  |
| Passeriformes: Estrildidae | Black-faced Waxbill | *Estrilda erythronotos* |  |  |  |
| Passeriformes: Estrildidae | Blue Waxbill | *Uraeginthus angolensis* |  | 4 |  |
| Passeriformes: Estrildidae | Bronze Mannikin | *Spermestes cucullata* | 8 | 4 |  |
| Passeriformes: Estrildidae | Common Waxbill | *Estrilda astrild* | 7 | 4 |  |
| Passeriformes: Estrildidae | Cut-throat Finch | *Amadina fasciata* |  |  |  |
| Passeriformes: Estrildidae | Green-backed Twinspot | *Mandingoa nitidula* |  |  |  |
| Passeriformes: Estrildidae | Green-winged Pytilia | *Pytilia melba* |  | 2 |  |
| Passeriformes: Estrildidae | Jameson's Firefinch | *Lagonosticta rhodopareia* |  |  |  |
| Passeriformes: Estrildidae | Pink-throated Twinspot | *Hypargos margaritatus* |  |  |  |
| Passeriformes: Estrildidae | Red-billed Firefinch | *Lagonosticta senegala* |  |  |  |
| Passeriformes: Estrildidae | Red-headed Finch | *Amadina erythrocephala* |  |  |  |
| Passeriformes: Estrildidae | Swee Waxbill | *Coccopygia melanotis* |  |  |  |
| Passeriformes: Estrildidae | Violet-eared Waxbill | *Granatina granatina* |  |  |  |
| Passeriformes: Estrildidae | Zebra Waxbill | *Amandava subflava* |  |  |  |
| Passeriformes: Fringillidae | Forest Canary | *Crithagra scotops* |  |  |  |
| Passeriformes: Fringillidae | Lemon-breasted Canary | *Crithagra citrinipectus* |  |  |  |
| Passeriformes: Fringillidae | Yellow-fronted Canary | *Crithagra mozambica* | 8 | 8 |  |
| Passeriformes: Hyliotidae | Southern Hyliota | *Hyliota australis* |  |  |  |
| Passeriformes: Laniidae | Lesser Grey Shrike | *Lanius minor* |  |  |  |
| Passeriformes: Laniidae | Magpie Shrike | *Urolestes melanoleucus* |  | 3 |  |
| Passeriformes: Laniidae | Red-backed Shrike | *Lanius collurio* |  | 2 |  |
| Passeriformes: Laniidae | Southern Fiscal | *Lanius collaris* |  | 4 |  |
| Passeriformes: Laniidae | White-crowned Shrike | *Eurocephalus anguitimens* |  |  |  |
| Passeriformes: Leiothrichidae | Arrow-marked Babbler | *Turdoides jardineii* |  | 10 |  |
| Passeriformes: Leiothrichidae | Southern Pied Babbler | *Turdoides bicolor* |  |  |  |
| Passeriformes: Locustellidae | Little Rush Warbler | *Bradypterus baboecala* |  | 1 |  |
| Passeriformes: Macrosphenidae | Cape Grassbird | *Sphenoeacus afer* |  |  |  |
| Passeriformes: Macrosphenidae | Long-billed Crombec | *Sylvietta rufescens* | 2 | 2 |  |
| Passeriformes: Malaconotidae | Black-backed Puffback | *Dryoscopus cubla* |  | 10 |  |
| Passeriformes: Malaconotidae | Black-crowned Tchagra | *Tchagra senegalus* |  | 2 |  |
| Passeriformes: Malaconotidae | Brown-crowned Tchagra | *Tchagra australis* |  |  |  |
| Passeriformes: Malaconotidae | Brubru | *Nilaus afer* |  |  |  |
| Passeriformes: Malaconotidae | Gorgeous Bush-shrike | *Telophorus viridis* |  |  |  |
| Passeriformes: Malaconotidae | Grey-headed Bush-shrike | *Malaconotus blanchoti* | 3 | 5 |  |
| Passeriformes: Malaconotidae | Olive Bush-shrike | *Chlorophoneus olivaceus* |  | 3 |  |
| Passeriformes: Malaconotidae | Orange-breasted Bush-shrike | *Chlorophoneus sulfureopectus* |  | 2 |  |
| Passeriformes: Malaconotidae | Southern Boubou | *Laniarius ferrugineus* |  |  |  |
| Passeriformes: Malaconotidae | Tropical Boubou | *Laniarius aethiopicus* |  |  |  |
| Passeriformes: Monarchidae | African Paradise-flycatcher | *Terpsiphone viridis* |  | 1 |  |
| Passeriformes: Monarchidae | Blue-mantled Paradise-flycatcher | *Trochocercus cyanomelas* |  |  |  |
| Passeriformes: Motacillidae | African Pipit | *Anthus cinnamomeus* |  |  |  |
| Passeriformes: Motacillidae | African Pied Wagtail | *Motacilla aguimp* |  | 1 |  |
| Passeriformes: Motacillidae | Buffy Pipit | *Anthus vaalensis* |  |  |  |
| Passeriformes: Motacillidae | Bushveld Pipit | *Anthus caffer* |  |  |  |
| Passeriformes: Motacillidae | Cape Wagtail | *Motacilla capensis* |  |  |  |
| Passeriformes: Motacillidae | Plain-backed Pipit | *Anthus leucophrys* |  |  |  |
| Passeriformes: Motacillidae | Striped Pipit | *Anthus lineiventris* |  |  |  |
| Passeriformes: Motacillidae | Yellow-throated Longclaw | *Macronyx croceus* |  |  |  |
| Passeriformes: Muscicapidae | African Dusky Flycatcher | *Muscicapa adusta* |  |  |  |
| Passeriformes: Muscicapidae | African Stonechat | *Saxicola torquatus* |  |  |  |
| Passeriformes: Muscicapidae | Arnott's Chat | *Myrmecocichla arnotti* |  |  |  |
| Passeriformes: Muscicapidae | Ashy Flycatcher | *Muscicapa caerulescens* | 3 | 2 |  |
| Passeriformes: Muscicapidae | Brown Scrub Robin | *Cercotrichas signata* |  |  |  |
| Passeriformes: Muscicapidae | Cape Rock Thrush | *Monticola rupestris* |  |  |  |
| Passeriformes: Muscicapidae | Capped Wheatear | *Oenanthe pileata* |  |  |  |
| Passeriformes: Muscicapidae | Eastern Bearded Scrub Robin | *Cercotrichas quadrivirgata* |  | 2 |  |
| Passeriformes: Muscicapidae | Familiar Chat | *Oenanthe familiaris* |  |  |  |
| Passeriformes: Muscicapidae | Fiscal Flycatcher | *Sigelus silens* |  |  |  |
| Passeriformes: Muscicapidae | Grey Tit Flycatcher | *Myioparus plumbeus* |  |  |  |
| Passeriformes: Muscicapidae | Mocking Cliff Chat | *Thamnolaea cinnamomeiventris* |  |  |  |
| Passeriformes: Muscicapidae | Pale Flycatcher | *Bradornis pallidus* |  |  |  |
| Passeriformes: Muscicapidae | Red-capped Robin Chat | *Cossypha natalensis* |  | 2 |  |
| Passeriformes: Muscicapidae | Southern Black Flycatcher | *Melaenornis pammelaina* | 2 | 2 |  |
| Passeriformes: Muscicapidae | Spotted Flycatcher | *Muscicapa striata* |  |  |  |
| Passeriformes: Muscicapidae | White-browed Robin Chat | *Cossypha heuglini* |  | 2 |  |
| Passeriformes: Muscicapidae | White-browed Scrub Robin | *Cercotrichas leucophrys* |  | 2 |  |
| Passeriformes: Muscicapidae | White-throated Robin Chat | *Cossypha humeralis* |  |  |  |
| Passeriformes: Nectariniidae | Amethyst Sunbird | *Chalcomitra amethystina* |  | 4 |  |
| Passeriformes: Nectariniidae | Collared Sunbird | *Hedydipna collaris* |  |  |  |
| Passeriformes: Nectariniidae | Purple-banded Sunbird | *Cinnyris bifasciatus* |  |  |  |
| Passeriformes: Nectariniidae | Scarlet-chested Sunbird | *Chalcomitra senegalensis* |  |  |  |
| Passeriformes: Nectariniidae | White-bellied Sunbird | *Cinnyris talatala* |  | 1 |  |
| Passeriformes: Nicatoridae | Eastern Nicator | *Nicator gularis* |  |  |  |
| Passeriformes: Oriolidae | African Golden Oriole | *Oriolus auratus* |  |  |  |
| Passeriformes: Oriolidae | Eastern Black-headed Oriole | *Oriolus larvatus* |  |  |  |
| Passeriformes: Paridae | Southern Black Tit | *Melaniparus niger* |  |  |  |
| Passeriformes: Passeridae | House Sparrow | *Passer domesticus* | 8 | 9 |  |
| Passeriformes: Passeridae | Southern Grey-headed Sparrow | *Passer diffusus* | 2 | 1 |  |
| Passeriformes: Passeridae | Yellow-throated Bush Sparrow | *Gymnoris superciliaris* |  |  |  |
| Passeriformes: Phylloscopidae | Willow Warbler | *Phylloscopus trochilus* |  |  |  |
| Passeriformes: Platysteiridae | Black-throated Wattle-eye | *Platysteira peltata* |  |  |  |
| Passeriformes: Platysteiridae | Cape Batis | *Batis capensis* |  |  |  |
| Passeriformes: Platysteiridae | Chinspot Batis | *Batis molitor* |  | 1 |  |
| Passeriformes: Ploceidae | Fan-tailed Widowbird | *Euplectes axillaris* |  |  |  |
| Passeriformes: Ploceidae | Lesser Masked Weaver | *Ploceus intermedius* |  |  |  |
| Passeriformes: Ploceidae | Red-billed Buffalo Weaver | *Bubalornis niger* |  | 1 |  |
| Passeriformes: Ploceidae | Red-billed Quelea | *Quelea quelea* |  |  |  |
| Passeriformes: Ploceidae | Red-collared Widowbird | *Euplectes ardens* |  |  |  |
| Passeriformes: Ploceidae | Southern Masked Weaver | *Ploceus velatus* | 2 | 1 |  |
| Passeriformes: Ploceidae | Southern Red Bishop | *Euplectes orix* | 1 | 1 |  |
| Passeriformes: Ploceidae | Spectacled Weaver | *Ploceus ocularis* | 1 | 1 |  |
| Passeriformes: Ploceidae | Village Weaver | *Ploceus cucullatus* | 1 | 1 |  |
| Passeriformes: Ploceidae | White-winged Widowbird | *Euplectes albonotatus* |  |  |  |
| Passeriformes: Ploceidae | Yellow Bishop | *Euplectes capensis* | 1 | 1 |  |
| Passeriformes: Ploceidae | Yellow-crowned Bishop | *Euplectes afer* |  |  |  |
| Passeriformes: Pycnonotidae | Dark-capped Bulbul | *Pycnonotus barbatus tricolor* | 5 | 12 | 1 |
| Passeriformes: Pycnonotidae | Sombre Greenbul | *Andropadus importunus* |  |  |  |
| Passeriformes: Pycnonotidae | Yellow-bellied Greenbul | *Chlorocichla flaviventris* |  |  |  |
| Passeriformes: Remizidae | Grey Penduline Tit | *Anthoscopus caroli* |  |  |  |
| Passeriformes: Sturnidae | Violet-backed Starling | *Cinnyricinclus leucogaster* | 1 | 1 |  |
| Passeriformes: Sturnidae | Burchell's Starling | *Lamprotornis australis* |  |  |  |
| Passeriformes: Sturnidae | Cape Starling | *Lamprotornis nitens* | 5 | 4 |  |
| Passeriformes: Sturnidae | Common Myna | *Acridotheres tristis* |  | 7 |  |
| Passeriformes: Sturnidae | Greater Blue-eared Starling | *Lamprotornis chalybaeus* | 10 | 17 | 1 |
| Passeriformes: Sturnidae | Meves's Long-tailed Starling | *Lamprotornis mevesii* |  | 1 |  |
| Passeriformes: Sturnidae | Red-winged Starling | *Onychognathus morio* |  |  |  |
| Passeriformes: Sturnidae | Wattled Starling | *Creatophora cinerea* |  | 1 |  |
| Passeriformes: Sylviidae | Common Whitethroat | *Curruca communis* |  |  |  |
| Passeriformes: Sylviidae | Garden Warbler | *Sylvia borin* |  |  |  |
| Passeriformes: Turdidae | Groundscraper Thrush | *Psophocichla litsitsirupa* |  |  |  |
| Passeriformes: Turdidae | Kurrichane Thrush | *Turdus libonyana* |  |  |  |
| Passeriformes: Vangidae | Retz's Helmet-shrike | *Prionops retzii* |  |  |  |
| Passeriformes: Vangidae | White-crested Helmet-shrike | *Prionops plumatus* | 6 | 14 |  |
| Passeriformes: Viduidae | Broad-tailed Paradise Whydah | *Vidua obtusa* |  |  |  |
| Passeriformes: Viduidae | Dusky Indigobird | *Vidua funerea* |  |  |  |
| Passeriformes: Viduidae | Eastern Paradise Whydah | *Vidua paradisaea* |  |  |  |
| Passeriformes: Viduidae | Pin-tailed Whydah | *Vidua macroura* |  |  |  |
| Passeriformes: Viduidae | Purple Indigobird | *Vidua purpurascens* |  |  |  |
| Passeriformes: Viduidae | Shaft-tailed Whydah | *Vidua regia* |  |  |  |
| Passeriformes: Viduidae | Village Indigobird | *Vidua chalybeata* |  |  |  |
| Passeriformes: Zosteropidae | African Yellow White-eye | *Zosterops senegalensis* |  |  |  |
| Passeriformes: Zosteropidae | Cape White-eye | *Zosterops virens* |  | 2 |  |
| Total |  |  | 84 | 203 | 5 |
